# Detection of group A *Streptococcus *in tonsils from pediatric patients reveals high rate of asymptomatic streptococcal carriage

**DOI:** 10.1186/1471-2431-12-3

**Published:** 2012-01-09

**Authors:** Amity L Roberts, Kristie L Connolly, Daniel J Kirse, Adele K Evans, Katherine A Poehling, Timothy R Peters, Sean D Reid

**Affiliations:** 1 Department of Microbiology and Immunology, Wake Forest University School of Medicine, Winston-Salem, NC, USA; 2 Department of Otolaryngology-Head and Neck Surgery, Wake Forest University School of Medicine, Winston-Salem, NC, USA; 3 Department of Pediatrics, Wake Forest University School of Medicine, Winston-Salem, NC, USA; 4 Department of Epidemiology and Prevention, Wake Forest University School of Medicine, Winston-Salem, NC, USA

## Abstract

**Background:**

Group A *Streptococcus *(GAS) causes acute tonsillopharyngitis in children, and approximately 20% of this population are chronic carriers of GAS. Antibacterial therapy has previously been shown to be insufficient at clearing GAS carriage. Bacterial biofilms are a surface-attached bacterial community that is encased in a matrix of extracellular polymeric substances. Biofilms have been shown to provide a protective niche against the immune response and antibiotic treatments, and are often associated with recurrent or chronic bacterial infections. The objective of this study was to test the hypothesis that GAS is present within tonsil tissue at the time of tonsillectomy.

**Methods:**

Blinded immunofluorescent and histological methods were employed to evaluate palatine tonsils from children undergoing routine tonsillectomy for adenotonsillar hypertrophy or recurrent GAS tonsillopharyngitis.

**Results:**

Immunofluorescence analysis using anti-GAS antibody was positive in 11/30 (37%) children who had tonsillectomy for adenotonsillar hypertrophy and in 10/30 (33%) children who had tonsillectomy for recurrent GAS pharyngitis. Fluorescent microscopy with anti-GAS and anti-cytokeratin 8 & 18 antibodies revealed GAS was localized to the tonsillar reticulated crypts. Scanning electron microscopy identified 3-dimensional communities of cocci similar in size and morphology to GAS. The characteristics of these communities are similar to GAS biofilms from *in vivo *animal models.

**Conclusion:**

Our study revealed the presence of GAS within the tonsillar reticulated crypts of approximately one-third of children who underwent tonsillectomy for either adenotonsillar hypertrophy or recurrent GAS tonsillopharyngitis at the Wake Forest School of Medicine.

**Trial Registration:**

The tissue collected was normally discarded tissue and no patient identifiers were collected. Thus, no subjects were formally enrolled.

## Background

Group A *Streptococcus *(GAS) is a β-hemolytic, Gram-positive human pathogen capable of causing a wide variety of human disease. GAS is one of the predominant causes of acute bacterial tonsillopharyngitis [1-6]. Tonsillopharyngitis is an acute infection of the palatine tonsils and pharynx often presenting symptomatically with a sore throat, fever and cervical lymphadenopathy [7]. Patients diagnosed with GAS tonsillopharyngitis are prescribed antibiotic therapy to avoid the potential development of post-infectious sequelae such as acute rheumatic fever and acute rheumatic heart disease [1-6].

Prevention of rheumatic fever with antibacterial therapy can be life-saving, so it is important to identify patients with GAS pharyngitis. Because accurate clinical differentiation between viral and GAS pharyngitis is not possible, laboratory confirmation of GAS pharyngitis is recommended for children [8]. A common clinical problem occurs when patients frequently present with episodes of acute viral pharyngitis, but GAS is repeatedly detected by throat culture or antigen detection methods because some of these children may be chronic carriers of GAS. Approximately 20% of school-age children are estimated to be chronic carriers of GAS, defined as prolonged persistence of GAS without evidence of infection or an immune response [9]. Although chronic carriage is well known and widespread, it is poorly understood and its clinical relevance is unclear.

Antibacterial therapy sufficient to treat GAS pharyngitis and prevent acute rheumatic fever is not effective in eradicating GAS carriage [10,11]. There are a number of hypotheses proposed to explain chronic GAS carriage. 1) Intracellular survival of GAS in tonsillar epithelium has been reported [12,13]. 2) Non-GAS organisms present in the pharynx that produce beta-lactamases may confer antibacterial resistance to otherwise susceptible GAS by proximity. 3) Carriage may occur due to an absence of normal oral flora that inhibit GAS [14].

We have shown that GAS forms biofilms *in vitro *and *in vivo *[15,16]. As put forth by Donlan and Costerton, a biofilm is a bacterial sessile community encased in a matrix of extracellular polymeric substances and attached to a substratum or interface [17]. Biofilms are inherently tolerant to host defenses and antibiotic therapies and often involved in chronic or recurrent illness due to impaired clearance [18,19]. It is estimated that upwards of 60% of all bacterial infections involve biofilms including dental caries, periodontitis, otitis media, chronic tonsillitis, endocarditis, necrotizing fasciitis and others [17,18,20]. Recently, bacterial biofilms have been shown on the tonsillar surface although the colonizing organism(s) has not been identified [21].

We sought to test the hypothesis that GAS biofilms are present on pediatric tonsil samples after tonsillectomy thereby contributing to persistence of the organism. This study involved examination of the tonsillar reticulated crypt epithelium, which is a branching network throughout the palatine tonsil that increases surface area and functions to allow more efficient antigen sampling [22-24]. We used immunofluorescence to demonstrate the presence of GAS within the reticulated crypts of tonsils recovered from pediatric patients undergoing tonsillectomy for recurrent GAS infection or adenotonsillar hypertrophy (ATH). Scanning electron microscopy and Gram-staining confirmed the presence of biofilms of Gram-positive cocci on the surface of and within tonsils recovered from both pediatric populations (recurrent GAS tonsillopharyngitis and ATH) which had tested positive for GAS by immunohistochemistry.

## Methods

This study was approved by the Wake Forest University Health Sciences Institutional Review Board. We analyzed specimens of tonsils from children 2-18 years of age undergoing tonsillectomy for management of either adenotonsillar hypertrophy (ATH) or recurrent GAS infections in 2009-2010. Upon removal, tonsils were placed in sterile PBS and kept at 4°C until processing. One tonsil per child was prepared for immunofluorescence staining and three IF-positive samples underwent scanning electron microscopy and tissue Gram-staining. Clinical information without personal identifiers was collected on a standardized form. It should be noted that we did not have access to samples from patients not requiring tonsillectomy. Thus, the cohort is biased and findings may not be applicable to pediatric GAS carriers that do not require such surgery.

### Immunofluorescence

#### Processing

One palatine tonsil per child was fresh frozen in OCT resin (Sakura Finetek, Torrance, CA) within a peel-a-way disposable plastic tissue embedding mold (Polysciences, Inc., Warrington, PA) and stored at -80°C. Samples were acclimated to -20°C, cut into 10 μm sections with a cryotome, placed onto positively charged microscope slides (Fisher Scientific, Fair Lawn, NJ), and stored at -20°C until immunofluorescence staining. For immunofluorescence staining, the slides were brought to room temperature, briefly fixed with 4% paraformaldehyde-PBS (PFMA)(Sigma-Aldrich, St. Louis, MO), and blocked for 30 min with 1% bovine serum albumin (BSA)(Amresco, Solon, OH) to control for non-specific antibody staining prior to addition of primary antibodies at a 1 to 500 dilution.

#### Group *A Streptococcus*

Individual sections were stained with primary rabbit anti-*Streptococcus *group A IgG (anti-GAS) (US Biological, Swampscott, MA, #S7974-28) in 1% BSA-PBS for 30 min in a 37°C incubator. While the company certified that the antibody does not react with other streptococcal groups (including groups C, F and G), our own testing confirmed the antibody did not cross-react with group B *Streptococcus*, viridans group *Streptococcus *nor *Streptococcus pneumoniae *(Tigr4) (data not shown). This anti-GAS antibody has been successfully used for immunofluorescence previously by our group [16].

#### Tonsillar crypt epithelial

Cytokeratin 8 & 18 are co-expressed specifically by tonsillar crypt epithelial cells [22,25]. Individual sections were stained with primary mouse monoclonal anti-Cytokeratin 8 and Cytokeratin 18 (Thermo Scientific, Fremont, CA) in 1% BSA-PBS for 30 min in a 37°C incubator.

#### Analysis

Individual sections were concurrently stained with antibody for GAS and for cytokeratin 8 & 18 to identify the presence of GAS and determine if it was localized to the tonsillar crypt epithelial cells. To control for autofluorescence and non-specific antibody staining, adjacent slides were either left unprobed with antibody or were probed with a rabbit anti-*Borrelia burgdorferi *IgG, (US Biological, Swampscott, MA) to control for IgG cross-reactivity and non-specific binding. As a positive control, slides of GAS *in vivo *biofilm sections collected from infected animals from a separate study [16] were stained with rabbit anti-GAS. Secondary antibodies ((goat anti-rabbit IgG-Alexa 568) (Invitrogen Molecular Probes, Eugene, OR) and goat anti-mouse-IgG-Alexa 488 (Invitrogen Molecular Probes, Eugene, OR)) were applied and samples were incubated for 30 min in a 37°C incubator. Samples were coated with ProLong Gold antifade reagent (Invitrogen Molecular Probes, Eugene, OR). Specific identification of GAS within the IF stained tonsil material was primarily visualized using a Nikon Eclipse TE300 Light Microscope equipped with an EXFO Xcite 120 Illumination System (Nikon Instruments Inc., Melville, NY) with a QImaging Retiga-EXi camera (AES, Perth, Australia) and Image J software.

### Gram-staining

Three tonsils that were positive for GAS by immunofluorescence analysis also underwent analysis by Gram-staining. Adjacent slides to those positive for immunofluorescence were Gram-stained using the Taylor's Brown-Brenn modified Gram-stain procedure. Samples were analyzed with a Nikon Eclipse TE300 Light Microscope (Nikon Instruments, Inc., Melville, NY). Images were taken using a QImaging Retiga-EXi camera (AES, Perth, Australia) and stored through Image J software.

### Scanning electron microscopy

A portion of three immunofluorescence-positive GAS tonsils from each group were fixed for 1 hour with 2.5% glutaraldehyde-PBS and then rinsed twice for 10 minutes in PBS prior to dehydration in a graded ethanol series. The samples were then subjected to critical point drying, mounted onto stubs, and sputter coated with palladium prior to viewing with a Philips SEM-515 scanning electron microscope (FEI, Hillsboro, OR). As a positive example of GAS biofilm formation on the surface of tissue, excised skin epithelium from pigs colonized *ex vivo *with GAS was processed and viewed as described above.

### Statistical Analysis

Categorical variables were analyzed by chi-square or Fisher's exact tests. A *P*-value of < 0.05 was considered statistically significant. Stata 8.1 (Stata Corporation, College Station, TX) was used for all analyses.

## Results

### Characteristics of study population undergoing tonsillectomy

The children undergoing tonsillectomy ranged in age from 2 years to 18 years with over half the children being 5-13 years of age. The age groups, gender, and race/ethnicity of children undergoing tonsillectomy for recurrent GAS tonsillopharyngitis or for ATH were similar (Table 1). Children undergoing tonsillectomy for recurrent GAS tonsillopharyngitis were more likely to have had a diagnosis of streptococcal pharyngitis in the prior year and history of ear tube placement than those with ATH.

**Table 1:** Characteristics of study population undergoing tonsillectomy

^**a **^**Characteristic**	Recurrent GAS pharyngitis	Adenotonsillar hypertrophy	^**b**^***P*****-value**
Age group			0.67
< 5 yrs	8 (27%)	11 (38%)	
5-13 yrs	19 (63%)	16 (55%)	
> 13 yrs	3 (10%)	2 (7%)	

Gender			0.51
% Female	14 (47%)	16 (55%)	
% Male	16 (53%)	13 (45%)	

Race/Ethnicity			0.10
White	22 (76%)	17 (55%)	
Black	5 (17%)	8 (26%)	
Hispanic	1 (3%)	6 (19%)	
Black and White	1 (3%)	0 (0%)	

GAS pharyngitis diagnosed within the past 12 months			< 0.001
Yes	30 (100%)	5 (17%)	
No	0 (0%)	24 (83%)	

History of ear tubes			0.04
Yes	9 (31%)	2 (7%)	
No	20 (69%)	27 (93%)	

^a ^There are 1 or 2 missing values for each category.

^b ^Statistical Analysis. Categorical variables were analyzed by chi-square or Fisher's exact tests. A *P*- value of < 0.05 was considered statistically significant. Stata 8.1 (Stata Corporation, College Station, TX) was used for all analyses.

### Prevalence of GAS in pediatric tonsils after tonsillectomy

Overall, 21 (35%) of 60 tonsils were positive for GAS by immunofluorescence. The proportion of tonsil samples that had GAS detected by immunofluorescence with or without acute symptoms of streptococcal pharyngitis was similar for children undergoing surgery for ATH (11 (37%) of 30 samples) and for those with recurrent GAS infection (10 (33%) of 30 samples, *P *= 0.79). Importantly, the anti-GAS antibody used does not cross-react with other streptococcal groups (US Biological, Swampscott, MA, #S7974-28).

### Detection of GAS within the tonsillar crypts

**Figure 1:**
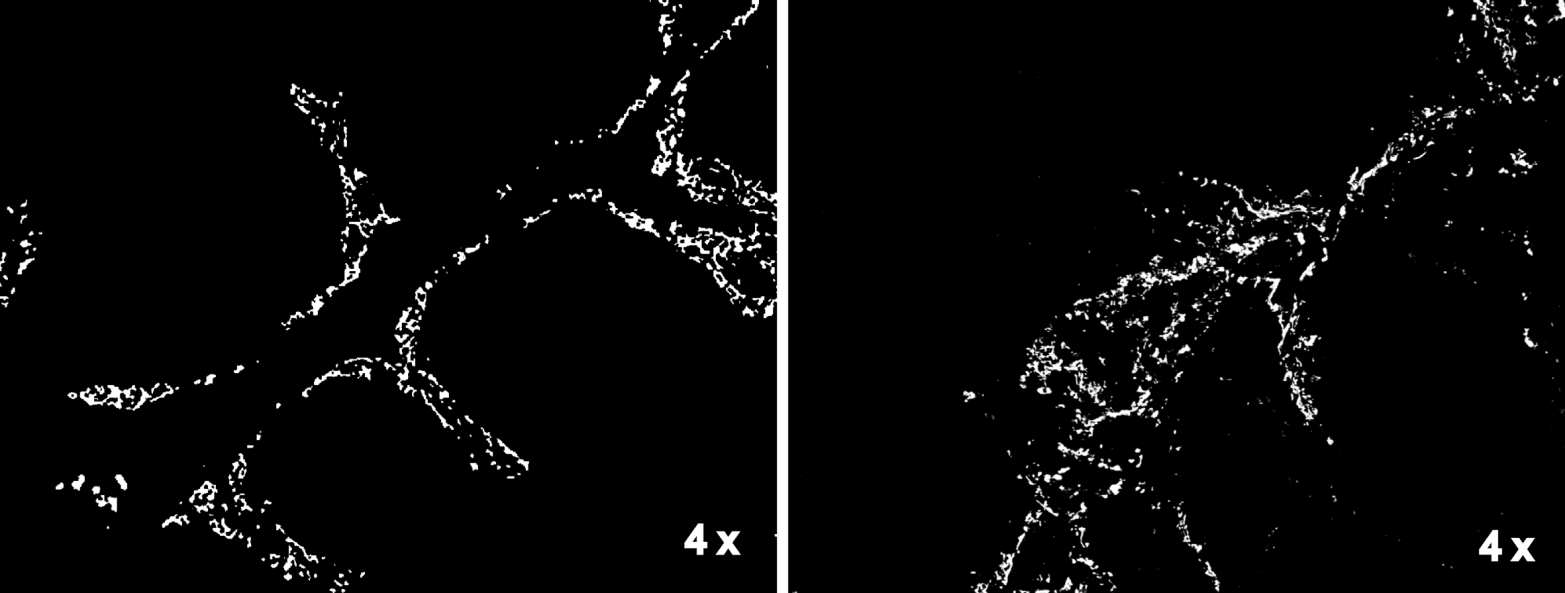
**Fluorescent antibody staining (10 μm sections) of Cytokeratin 8 & 18 (white) allows visualization of the tonsillar crypt epithelium**. Representative images of tonsils removed due to ATH (left) or recurrent GAS infection (right) are shown at 4 × magnification.

We hypothesized that the detected GAS was localized in the tonsillar crypts. Given that the reticulated crypt epithelium expresses unique cytokeratin markers 8 & 18, we elected to use dual staining immunofluorescence in an effort to detect the colocalization of crypt markers with GAS [22,25]. As proof of principle, the branching network of the crypts was readily visible following staining with anti-cytokeratin 8 and anti-cytokeratin 18 (Figure 1).

**Figure 2:**
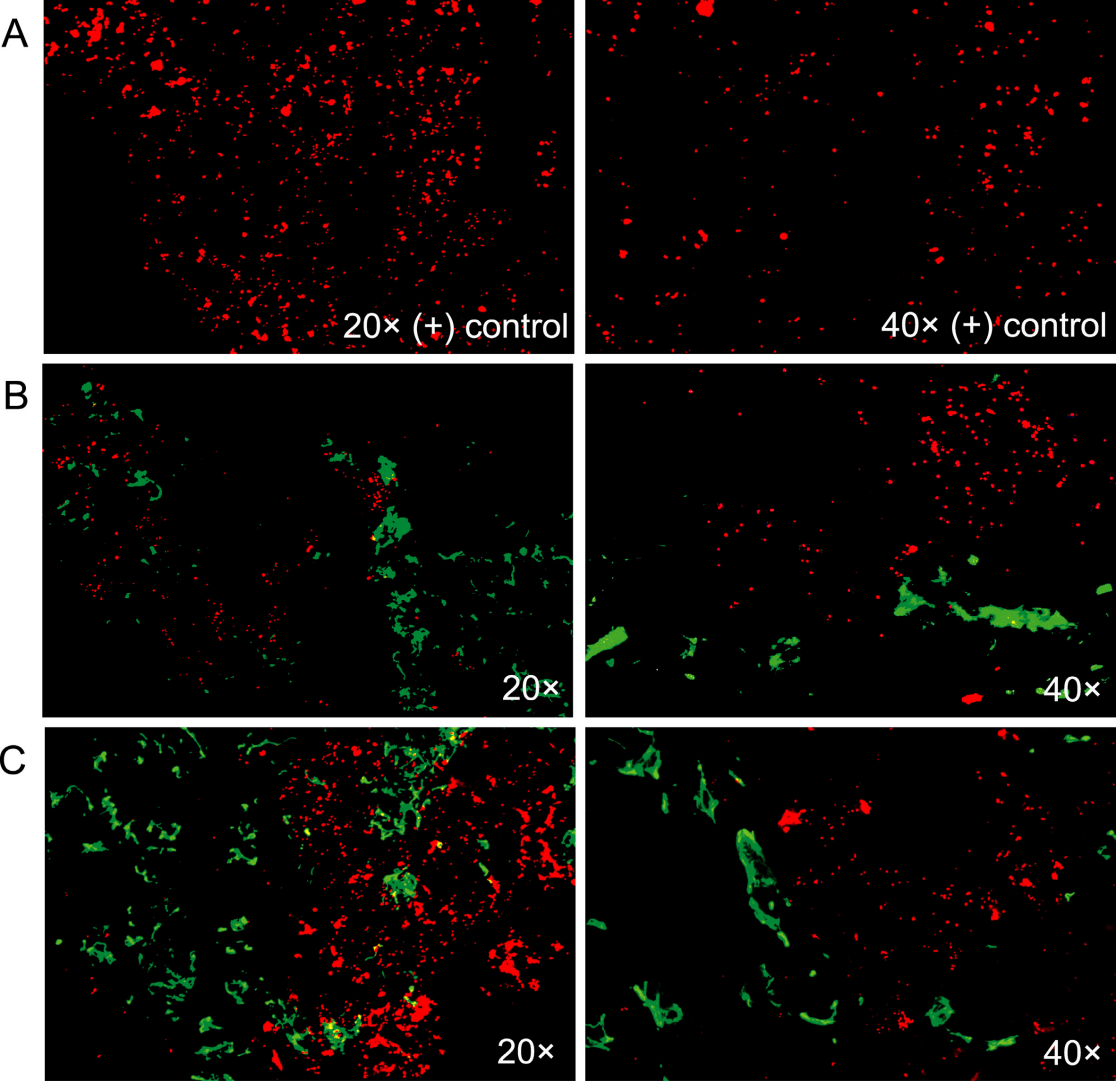
**Fluorescent antibody staining of GAS (red) within the crypts (green) of pediatric tonsils removed due to recurrent GAS tonsillopharyngitis**. **(A) **GAS within *in vivo *biofilm from a chinchilla. **(B **and **C) **GAS within the tonsillar crypts.

**Figure 3:**
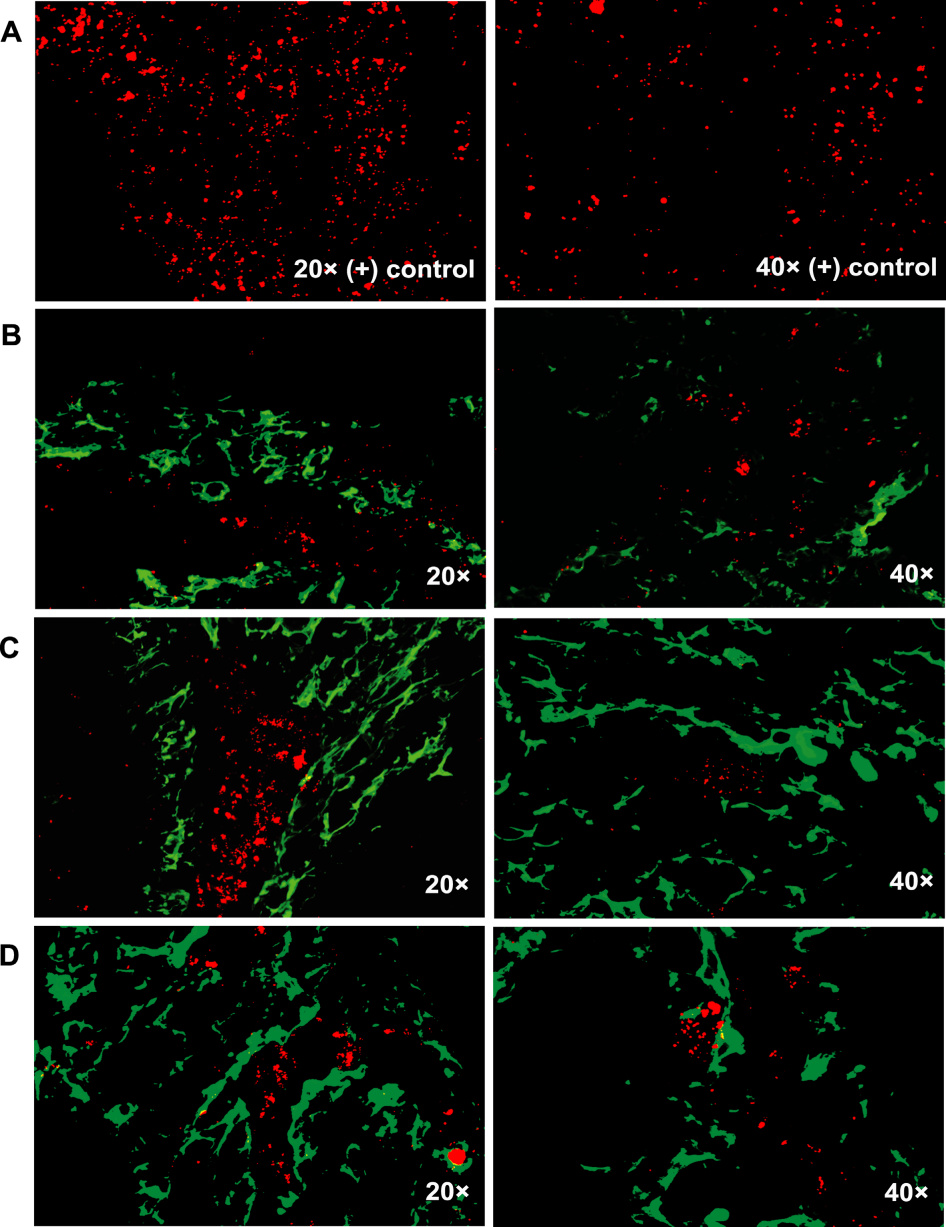
**Fluorescent antibody staining of GAS (red) within the crypts (green) of pediatric tonsils removed due to ATH**. (**A) **GAS within *in vivo *biofilm from a chinchilla. (**B**, **C, D) **GAS within the tonsillar crypts.

Dual staining immunofluorescence revealed that GAS (RED) consistently localized to the tonsillar reticulated crypt epithelium (GREEN) in both samples from recurrent GAS infection (Figure 2. row B, C) and ATH (Figure 3. row B, C, D) cases. Representative images of GAS localization are shown (Figure 2, Figure 3). Positive controls for the detection of GAS consisted of *in vivo *biofilm samples collected from a chinchilla middle ear model of GAS infection (Figure 2 and 3. row A) [16].

### SEM detection of bacterial biofilms on the tonsillar surface of samples positive for the presence of GAS

**Figure 4:**
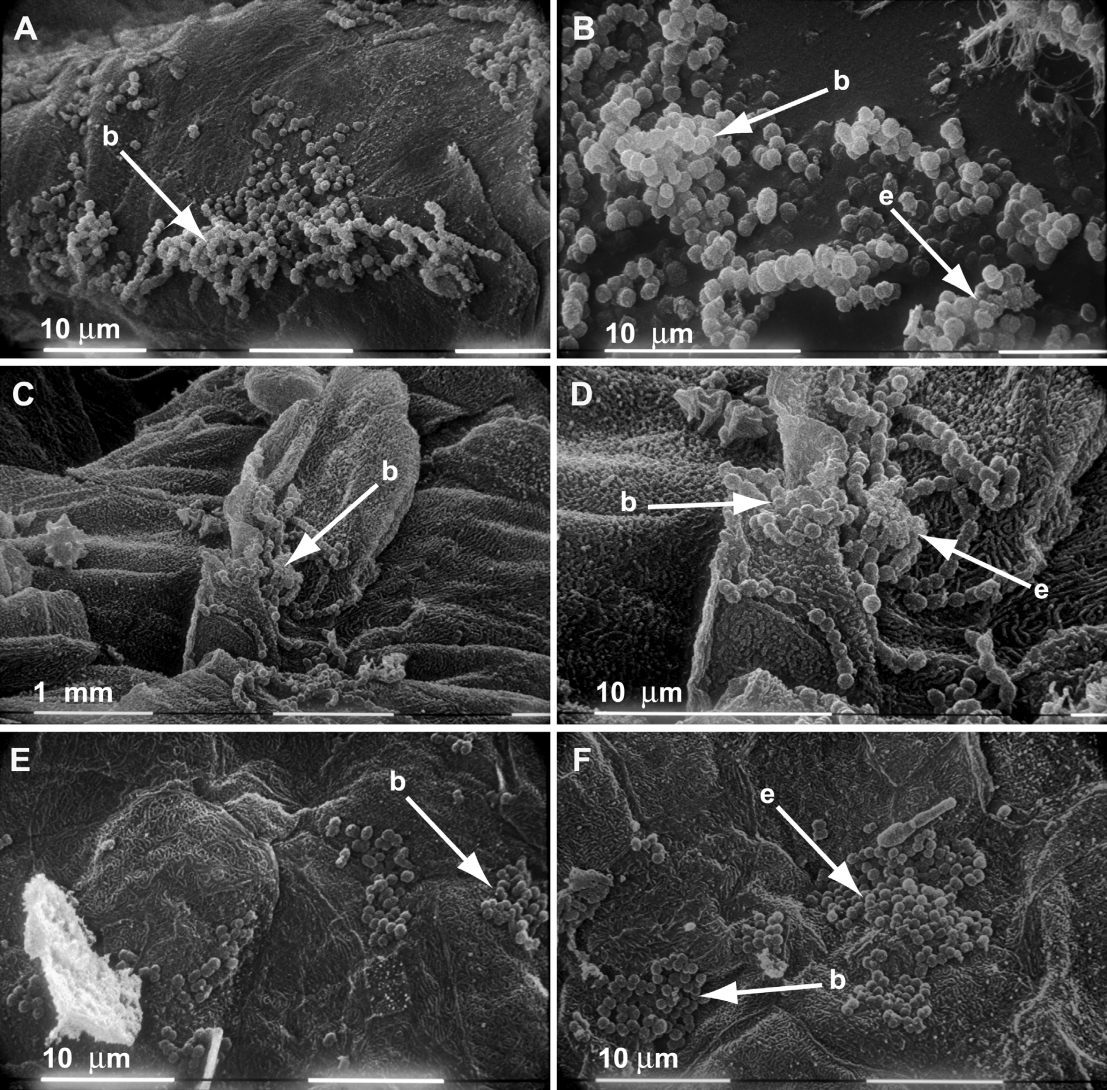
**SEM showing chains of adherent cocci organized into biofilms attached to the surface of pig skin epithelium (**A **and **B**) and to the surface of a tonsil removed due to recurrent GAS infection (**C **and **D**) or ATH (**E **and **F**)**.

SEM was utilized to identify the presence of 3-dimensional bacterial communities on the tonsillar surface that are indicative of biofilms. As a positive control, we used an established model in our laboratory of growing GAS biofilms on pig skin. This model allows the development of biofilms that are identical in appearance to those we have observed in material recovered from GAS infected middle ears of chinchillas and GAS infected soft tissue of mice [16]. Excised skin epithelium from pigs was incubated in the presence of GAS for a period of 24 h and analyzed by SEM for the presence of biofilms. As expected, distinct, 3-dimensional communities of adherent chains of GAS cocci (biofilms) were observed on the epithelium surface (Figure 4A, B). Close inspection revealed the presence of extracellular matrix material within the makeup of the biofilm (Figure 4A, B). SEM revealed homologous biofilm structures on the surface of tonsils that had previously tested positive for the presence of GAS by immunofluorescence (Figure 4C-F). As in our positive controls, biofilms were not evenly distributed across the tonsillar surface but relegated to folds in the tonsil epithelium. While the size of the biofilms differed from sample to sample, the overall morphology of the structures observed were consistent among samples from recurrent GAS infection (Figure 4C, D) and ATH (Figure 4E, F). Close inspection revealed the presence of extracellular matrix associated with the biofilms. We interpret this data as evidence of GAS biofilms on the tonsil surface.

### Gram-staining was utilized to provide further evidence of Gram-positive biofilm formation

**Figure 5:**
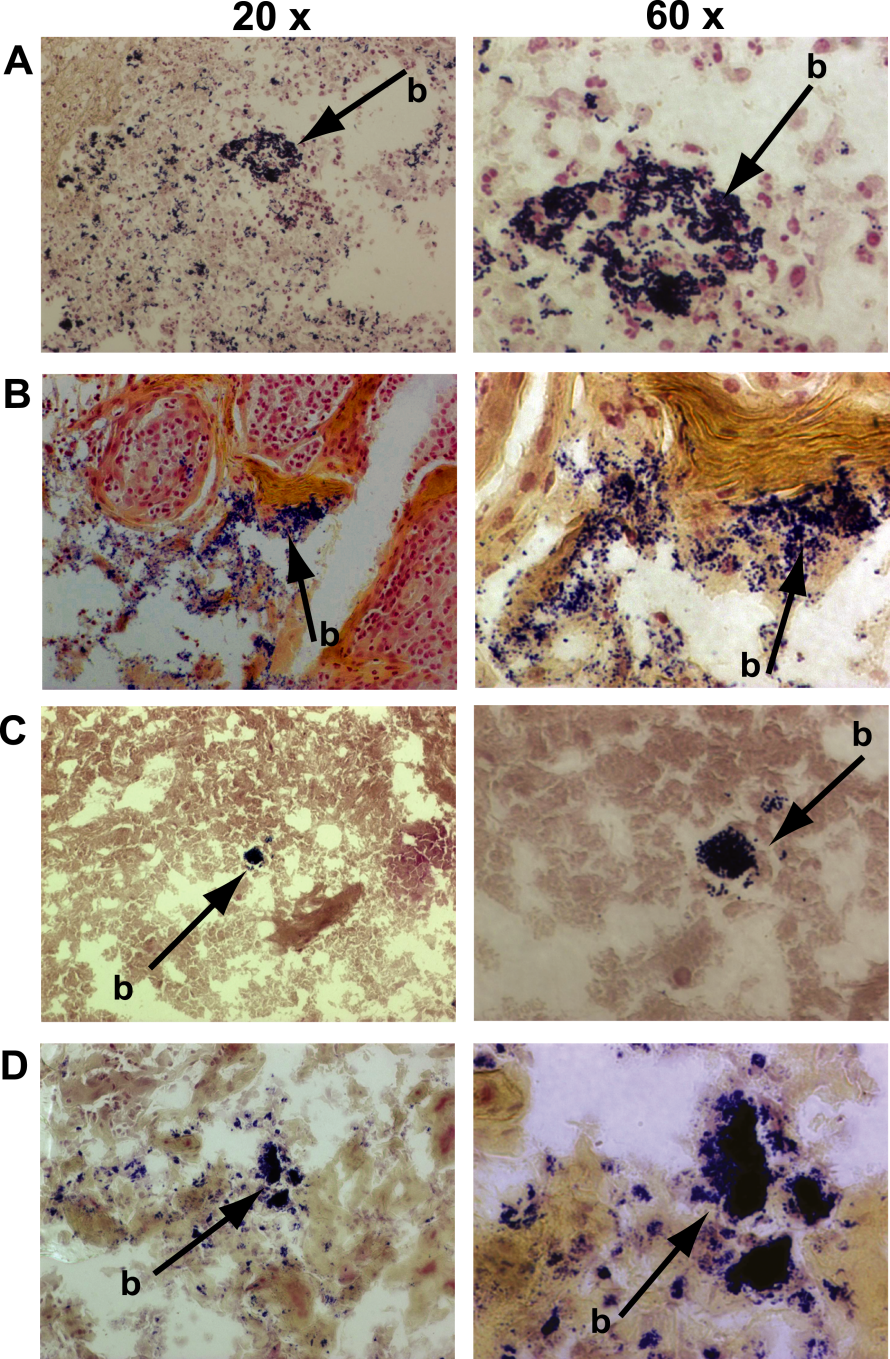
**Gram-stain showing the positive detection of GAS biofilm (b) in a chinchilla sample (**A**)**. Detection of a Gram-positive biofilm (b) in a tonsil removed due to recurrent GAS infection (**B**) or ATH (**C **and **D**).

We and others have shown that adherent, 3-dimensional microcolonies of bacteria within tissue visualized with Gram-staining are representative of the presence of biofilm [16,21,26,27]. As further evidence of GAS biofilms in the collected tissue, we next analyzed tonsillar tissue for the presence of microcolonies by way of Gram-staining. As a positive control for biofilm detection, we Gram-stained specimens collected from a chinchilla that was infected with GAS in the middle ear [16]. Biofilms of GAS were clearly evident in addition to dispersed GAS (Figure 5A). Homologous biofilms consisting of Gram-positive cocci were observed in tonsil specimens collected from children presenting with either recurrent GAS tonsillopharyngitis (Figure 5B) or ATH (Figure 5C, D).

## Discussion

In the present study, we sought to test the hypothesis that GAS was present within or on pediatric palatine tonsils and to see if we could identify evidence of biofilm formation. To achieve this, we analyzed surgically excised tonsils from 30 pediatric patients undergoing tonsillectomy due to recurrent GAS tonsillopharyngitis. Originally, we planned to examine a limited number of surgically excised tonsils from patients undergoing tonsillectomy for ATH as these patients were asymptomatic for GAS infection, and thus we thought these samples would provide a negative control for the detection of GAS. However, we readily detected the presence of GAS in these samples by immunofluorescence and scanning electron microscopy revealed the presence of biofilms made up of chains of cocci which morphologically resembled GAS. Given this result, we examined the tonsils excised from a total of 30 patients presenting with ATH in addition to the tonsils excised from 30 patients presenting with recurrent GAS tonsillopharyngitis.

We discovered that a similar proportion of tonsils from children with either ATH or recurrent GAS tonsillopharyngitis were positive for GAS by immunofluorescence (~35% positive). Of note, our method of immunofluorescence allowed the dual detection of both GAS and the cellular markers of the reticulated crypt epithelium, cytokeratin 8 & 18. By this method, we were able to show that the GAS detected had colonized throughout the tonsillar crypts in both sets of patient tonsil samples.

The finding that tonsillar tissue from children with ATH patients showed GAS in such a high percentage was unexpected. However, bacterial carriage by children with ATH is not unprecedented. Several previous studies of adenotonsillar hypertrophy have provided evidence that increased numbers of pathogenic bacteria can be recovered from homogenized hypertrophic tonsil cores compared to swabs of those tonsils alone [28,29]. Indeed, it is proposed that lymphoid hyperplasia (chronic enlargement) is correlated with increased bacterial load and increased B- and T-lymphocyte proliferation [30,31]. This phenomenon has been associated with a number of bacteria including *Staphyloccocus aureus*, *Haemophilus influenzae, S. pneumoniae*, as well as GAS [28]. However, a review of the literature revealed that the percentage of hypertrophic tonsils positive for GAS by Brodsky et al. (16%) and Stjernquist-Desatnik et al. (20%) was lower than the 36.7% positive that we observed [28,32]. Our method of detection is more sensitive (antibody based immunofluorescence vs. culture), but this difference in frequency of detection may also be due to geographic location or the fact that our study occurred almost 20 years after the reports referenced.

Our results support the hypothesis that GAS colonize pediatric palatine tonsils as a biofilm. SEM clearly reveals the presence of 3-dimensional communities of chains of cocci in tonsils that had tested positive for the presence of GAS by immunofluorescence. These structures closely resemble the *in vivo *GAS biofilms grown *ex vivo *on pig epithelium. Furthermore, Gram-staining reveals the presence of microcolonies of Gram-positive cocci indicative of biofilms in samples that tested positive for the presence of GAS. However, despite the fact that these samples were positive for GAS by immunofluorescence, we cannot rule out at this juncture that the biofilms observed by SEM or Gram-staining were not GAS.

Detailed information regarding antibiotic exposure prior to tonsillectomy was not collected; however, it is known that 100% of the children undergoing tonsillectomy for recurrent GAS tonsillopharyngitis had experienced a recent GAS infection. The finding that GAS were detected in roughly equivalent percentages in these two patient groups is consistent with the hypothesis that biofilms may be important in carrier state antibacterial resistance.

The high rate of asymptomatic GAS colonization that is presented here also has implications regarding the utility of rapid antigen tests and cell culture. The question of what is a true positive vs. a clinical false positive, especially in light of the potential for co-colonization by a viral pathogen, is a difficult one. Now that we have established the ability to rapidly detect the presence of GAS within the tonsillar crypt, our emphasis will turn to the collection and typing of these strains, analysis of the frequency of their isolation, as well as an elucidation of what makes up the GAS biofilm structure and how it is regulated. Specifically, our findings contribute to an understanding of GAS tonsillar colonization. Developing the capacity to distinguish patients with GAS tonsillopharyngitis from those with GAS colonization, or those with GAS colonization and viral tonsillopharyngitis is a clinically important goal that could greatly reduce unnecessary antibiotic use. This work may ultimately contribute to the development of clinically useful methods for identifying patients with longstanding GAS colonization.

## Conclusions

Our study revealed the presence of GAS within the tonsillar reticulated crypts of approximately one-third of children who underwent tonsillectomy for either adenotonsillar hypertrophy or recurrent GAS tonsillopharyngitis at the Wake Forest School of Medicine.

## Competing interests

The authors declare that they have no competing interests.

## Authors' contributions

ALR, KLC and SDR performed the analysis of the tonsil tissue while DJK and AKE performed the surgery to collect the tonsils. TRP and KAP assisted with analysis of patient data and helped write the Institutional Review Board protocol necessary to collect patient samples. All authors read and approved the final manuscript.

## Pre-publication history

The pre-publication history for this paper can be accessed here:

http://www.biomedcentral.com/1471-2431/12/3/prepub
